# Inflammatory potential of diet and risk of incident knee osteoarthritis: a prospective cohort study

**DOI:** 10.1186/s13075-020-02302-z

**Published:** 2020-09-10

**Authors:** Qiang Liu, James R. Hebert, Nitin Shivappa, Jianjun Guo, Ke Tao, Chao Zeng, Guanghua Lei, Jianhao Lin, Yuqing Zhang

**Affiliations:** 1 Arthritis Clinic and Research Center, Peking University People’s Hospital, No.11 Xizhimen South Road, Xicheng District, Beijing, 100044 China; 2 Division of Rheumatology, Allergy and Immunology, Massachusetts General Hospital, Harvard Medical School, 55 Fruit St., Boston, MA 02114 USA; 3 Cancer Prevention and Control Program, University of South Carolina, Columbia, SC USA; 4 Department of Epidemiology and Biostatistics, Arnold School of Public Health, University of South Carolina, Columbia, SC USA; 5 Capital University of Physical Education and Sports, Beijing, China; 6 Department of Orthopedics, Xiangya Hospital, Central South University, Changsha, China

**Keywords:** Osteoarthritis, Diet, Inflammation, Body mass index, Cohort

## Abstract

**Background:**

To examine the relation between inflammatory potential of diet and incident knee osteoarthritis (OA) and the role of BMI in the association of interest.

**Methods:**

In the Osteoarthritis Initiative, the energy-adjusted dietary inflammatory index (E-DII™) scores were calculated based on the Block Brief 2000 Food Frequency Questionnaire and categorized into sex-specific quartiles. Outcomes were incident (1) radiographic knee OA (ROA) (i.e., a KL grade ≥ 2) and (2) symptomatic knee OA (SxOA) (i.e., a combination of frequent knee pain and ROA). We fitted generalized estimating equation models to examine the association between E-DII scores and incident knee OA. We performed mediation analyses to assess the potential mediation by BMI in the DII-OA relation.

**Results:**

Over a 48-month follow-up period, 232 and 978 knees developed ROA and SxOA, respectively. Compared with the lowest (most anti-inflammatory) E-DII quartile, the odds ratio (OR) of incident ROA for the highest (most pro-inflammatory) E-DII quartile was 1.73 (95% confidence interval (CI) 1.15 to 2.62, *P*_trend_ = 0.007). The corresponding OR for SxOA was 1.43 (95% CI 1.16 to 1.76, *P*_trend_ = 0.001). The DII-OA association was significantly mediated via BMI with an indirect effect of 1.08 (95% CI 1.04, 1.13) for ROA and 1.13 (95% CI 1.09, 1.16) for SxOA, accounting for 20.4% and 44.5% of the total effect, respectively.

**Conclusions:**

A higher inflammatory potential of diet increased the risk of knee OA. The association was significantly mediated via BMI. Targeting the inflammatory potential of diet may be beneficial to reduce the risk of knee OA.

## Background

Diet plays a fundamental role in preventing many chronic diseases [1, 2]. One of the mechanisms is through regulating inflammation [3–5]. Indeed, the inflammatory potential of diet has been shown to be associated with obesity [6, 7] and cardiovascular diseases [8, 9], two conditions well known to be related to chronic, systemic inflammation. Osteoarthritis (OA), a common form of arthritis that is pathologically associated with biomechanics and inflammation [10], is highly prevalent among the elderly and is a leading cause of disability [11, 12]. A limited number of risk factors for OA are modifiable [13]. Diet can be modified on a daily basis, and the evidence for its role in OA development is accumulating [14, 15]. Despite the role of the whole diet in determining health outcomes, a majority of studies have focused on single dietary components. Complex human diets consist of multiple components that interact with one another and exert inflammatory potential as a whole; thus, it is reasonable to use a summary measure to quantify the inflammatory potential of diet as a whole. In this sense, a dietary index that summarizes the functional effect of food has an advantage over examining single dietary components in the study of diet-disease associations [16]. To the best of our knowledge, no studies have used a summary measure that quantifies the inflammatory potential of diet to examine the association of diet with the risk of incident OA.

High body mass index (BMI) is a well-established risk factor for OA and is associated with both biomechanics and inflammation in OA pathology [10, 17]. Diet plays a central role in determining BMI. At its simplest, diet provides the energy intake side of the energy balance equation. Reflecting the nuanced way that diet can exert an effect on body mass, a higher inflammatory potential of diet as measured by the dietary inflammatory index (DII^®^) has been shown, prospectively, to increase the risk of weight gain and incident obesity [6]. Despite the interplay between diet, BMI, and OA, the role of BMI in the diet-OA association remains largely unexplored. There is evidence that BMI can mediate the association of diet with serum inflammatory markers on the basis of its inflammatory potential [18]. For example, a recent study showed that adjustment for BMI substantially attenuated the association of dietary fiber with the risk of symptomatic knee OA (SxOA) [19]. Taken together, these findings suggest that BMI may function as a mediator in the association between the inflammatory potential of diet and knee OA. However, such a hypothesis has not been formally tested yet.

To fill in these gaps of knowledge, we conducted a longitudinal cohort study using data from the Osteoarthritis Initiative (OAI). We aimed to examine (1) the association of inflammatory potential of diet with the risk of incident knee OA and (2) the mediation effect of BMI in the association of interest.

## Methods

### Study population

The OAI is a multi-center, longitudinal prospective cohort study. At baseline, 4796 US men and women (58.5%) age 47–79 years with, or at high risk of, knee OA were recruited from four sites: Columbus, OH; Providence, RI; Baltimore, MD; and Pittsburgh, PA. Annual radiographic assessments of knee OA were carried out until the 48-month OAI follow-up visit; therefore, in the present study, we followed the participants until 48 months.

### Baseline assessment of inflammatory potential of diet

Habitual dietary intake of nutrients and foods was estimated at baseline using a validated dietary assessment tool, the Block Brief 2000 Food Frequency Questionnaire (FFQ) [20]. For each dietary component, the frequency of consumption was reported according to nine predetermined categories ranging from “never” to “everyday” with illustrations of standard portion sizes. Dietary intake of each component and energy was calculated based on nutrient composition values determined from the US Department of Agriculture nutrient database [21].

The DII, developed by Cavicchia et al. [22] and updated by Shivappa et al. [23], is a literature-derived population-based scoring algorithm to assess the inflammatory potential of diet as a whole based on 45 food parameters. A higher DII score indicates a greater inflammatory potential of diet (i.e., pro-inflammatory effect). Details regarding the development and calculation process are available elsewhere [23]. The DII has been evaluated for validity in 27 studies and shown to predict inflammatory markers including C-reactive protein (CRP), interleukin (IL)-6, and tumor necrosis factor (TNF)-α [22, 24–27]. Using data collected by the FFQ in the OAI, we calculated the energy-adjusted DII (E-DII™) based on the intake of 24 dietary components, defined as DII score per 4184 kJ (1000 kcal) of energy [28]. Dietary components available in the OAI for calculating E-DII scores included vitamin B12, vitamin B6, β-carotene, caffeine, carbohydrate, cholesterol, fat, fiber, folic acid, iron, magnesium, monounsaturated fat acids, niacin, protein, polyunsaturated fatty acids, riboflavin, saturated fat acids, selenium, thiamin, vitamin A, vitamin C, vitamin E, vitamin D, and zinc.

### Assessment of incident knee OA outcomes

At baseline and at each annual follow-up visit, frequent knee pain was queried and defined as pain, aching, or stiffness for more than half the days of a month during the past 12 months. Participants obtained weight-bearing fixed-flexion posterior-anterior view radiographs of both knees. Central reading was carried out to assess Kellgren and Lawrence (KL) grade for each knee. Any disagreement as to whether the knee at any time point had radiographic OA was adjudicated by a panel of three experienced readers including the two primary readers and one other. We defined a knee as having incident radiographic knee OA (ROA) if it did not have ROA at baseline (i.e., K/L = 0 or 1) and developed ROA (i.e., K/L ≥ 2) over the follow-up time. Incident knee SxOA was defined as a new onset of a combination of frequent knee pain and ROA in the same knee during the follow-up period.

### Assessment of other covariates and BMI as a potential mediator

At baseline, all participants were queried for age, sex, race, educational attainment, annual income, and tobacco use. Physical activity was assessed using the Physical Activity Scale for the Elderly (PASE) capturing a broad spectrum of habitual physical activity and is summarized into a continuous score with higher scores indicating higher levels of physical activity. PASE was validated for the assessment of physical activity among older adults with knee pain and physical disability [29]. At the 12-month follow-up visit, body weight and height were measured in light clothing without shoes using calibrated devices. BMI was calculated using weight (kg) divided by the square of height (m). We used BMI assessed at the 12-month follow-up visit as the mediator in the current study.

### Statistical analysis

We categorized E-DII scores into quartiles for men and women separately to account for sex differences in dietary intake of nutrients and foods. We examined the association of E-DII with the risk of knee ROA (or SxOA) by comparing higher E-DII quartiles (Q2, Q3, and Q4) with the lowest quartile (Q1, reference category) using generalized estimating equations (GEE) to account for the correlation between two knees for each participant. In the base model (model 1), we adjusted for age, sex (men vs women), race (White vs non-White), and total energy intake (kcal/day). In model 2, we further adjusted for educational attainment (below college vs college or above), annual income (< 50,000 US$ vs ≥ 50,000 US$), tobacco use (non-smoker vs former and current smoker), and PASE score. We tested for linear trend using the median value of each quartile of E-DII score as a continuous variable in the regression model. We also conducted alternative analyses fitting E-DII as a continuous variable in model 1 and model 2. We conducted sensitivity analyses by counting total knee replacement due to knee OA during the follow-up period as incident knee ROA or SxOA.

**Fig. 1:**
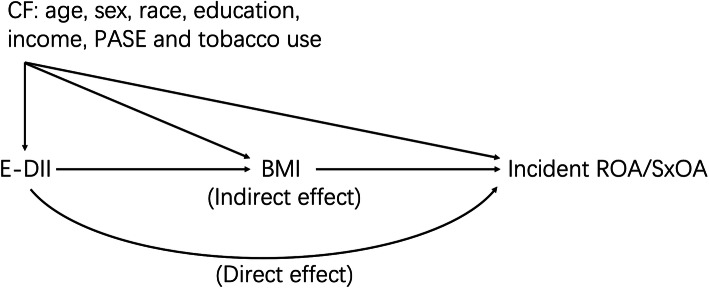
A directed acyclic graph for the decomposition of the total effect into direct and indirect effect. E-DII, energy-adjusted dietary inflammatory index; ROA, radiographic knee osteoarthritis; SxOA, symptomatic knee osteoarthritis; CF, confounder; PASE, Physical Activity Scale for the Elderly; BMI, body mass index

We performed mediation analyses to assess to what extent the association of E-DII score with incident ROA (or SxOA) was mediated through BMI assessed at the 12-month follow-up visit. In these analyses, we grouped the E-DII score into two categories using sex-specific median value as a cut-point. We decomposed the total effect of E-DII score on the risk of incident knee ROA (or SxOA) into two components [30, 31] (Fig. 1), i.e., (1) the indirect effect (or mediated effect) representing the effect of E-DII on the risk of incident knee ROA (or SxOA) mediated via BMI and (2) the direct effect representing the effect of E-DII score on the risk of incident knee ROA (or SxOA) that was not through BMI. We calculated the proportion of mediation to quantify the proportion of the effect of E-DII mediated by BMI [30, 31].

All statistical analyses were performed using Stata/SE 15.1 (StataCorp, TX, USA). A *P* value < 0.05 (two-sided) was considered statistically significant.

## Results

**Fig. 2:**
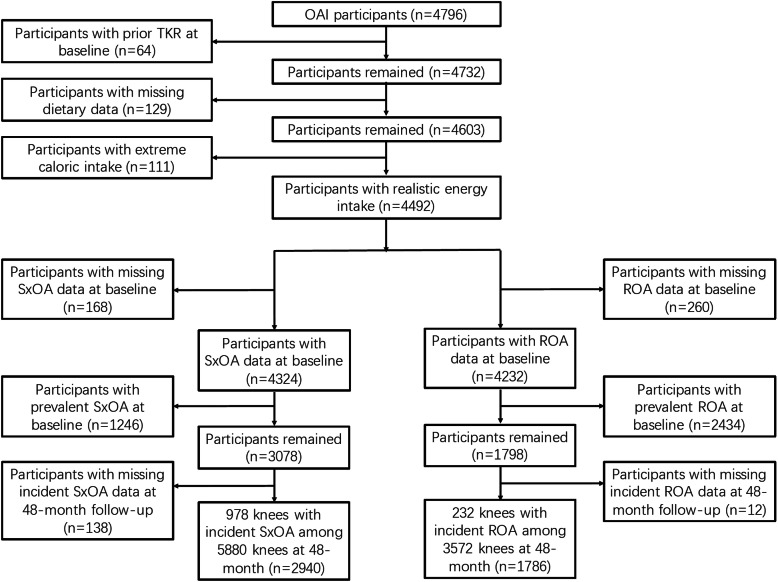
Osteoarthritis Initiative participants included in the final analyses until 48-month follow-up

We included 1786 and 2940 eligible participants in the final analyses for the association of E-DII score with incident knee ROA and SxOA, respectively. The inclusion of subjects and knees is described in Fig. 2.

We summarized the baseline characteristics according to sex-specific quartiles of E-DII score in Table 1. Among participants included in the analysis of incident ROA or SxOA, subjects in the highest E-DII quartile (Q4, reflecting the most pro-inflammatory diet) were younger, more likely to have attained college or higher educational level, and had an annual income ≥ 50,000 US$ as well as a higher total energy intake and BMI than their counterparts in the lowest quartile of E-DII (Q1, representing the most anti-inflammatory diet).

**Table 1:** Participants’ baseline characteristics according to quartiles of the energy-adjusted dietary inflammatory index in OAI

	Eligible participants for incident ROA				Eligible participants for incident SxOA			
	Q1 (*n* = 448)	Q2 (*n* = 446)	Q3 (*n* = 446)	Q4 (*n* = 446)	Q1 (*n* = 736)	Q2 (*n* = 735)	Q3 (*n* = 735)	Q4 (*n* = 734)
E-DII median in men (IQR)	− 3.5 (− 5.5, − 2.9)	− 2.1 (− 2.8, − 1.4)	− 0.7 (− 1.4, 0.3)	1.3 (0.3, 3.8)	− 3.5 (− 5.5, − 2.9)	− 2.1 (− 2.9, − 1.5)	− 0.7 (− 1.5, 0.3)	1.3 (0.3, 3.9)
E-DII median in women (IQR)	− 4.1 (− 5.6, − 3.5)	− 3.0 (− 3.5, − 2.4)	− 1.8 (− 2.4, − 0.9)	0.4 (− 0.9, 3.9)	− 4.1 (− 5.6, − 3.5)	− 3.0 (− 3.5, − 2.5)	− 1.9 (− 2.5, − 1.0)	0.1 (− 1.0, 3.9)
Age (years)*	61.0 ± 9.2	60.7 ± 9.1	58.9 ± 9.1	57.2 ± 8.4	62.8 ± 9.1	62.0 ± 9.2	61.1 ± 9.4	58.9 ± 8.7
Female, *n* (%)	259 (57.8)	258 (57.8)	258 (57.8)	258 (57.8)	431 (59.0)	431 (58.6)	431 (58.6)	430 (58.6)
Race (European-American), *n* (%)	385 (86.3)	384 (86.1)	390 (87.4)	351 (78.9)	636 (87.0)	631 (85.9)	633 (81.5)	597 (85.0)
Education, *n* (%)								
< College level	136 (30.4)	149 (33.4)	165 (37.0)	192 (43.1)	227 (31.0)	225 (30.6)	293 (39.9)	317 (43.2)
College level or above	312 (69.6)	297 (66.6)	281 (63.0)	254 (56.9)	509 (69.0)	510 (69.4)	442 (60.1)	417 (56.8)
Yearly income, *n* (%)								
< 50,000 US$	128 (30.5)	148 (34.6)	142 (33.6)	166 (39.6)	241 (35.0)	244 (34.9)	256 (36.7)	273 (39.6)
≥ 50,000 US$	292 (69.5)	280 (65.4)	281 (66.4)	253 (60.4)	448 (65.0)	455 (65.1)	442 (63.3)	417 (60.4)
Tobacco use, *n* (%)								
Non-smoker	361 (80.6)	343 (77.4)	345 (77.7)	360 (81.2)	591 (80.4)	574 (78.4)	576 (78.6)	584 (80.1)
Smoker	87 (19.4)	100 (22.6)	99 (22.3)	83 (18.8)	144 (19.6)	158 (21.6)	157 (21.4)	145 (19.9)
BMI (kg/m^2^)*	26.3 ± 4.1	27.6 ± 4.6	27.7 ± 4.3	27.8 ± 4.8	26.9 ± 4.3	27.9 ± 4.5	28.2 ± 4.5	28.8 ± 4.9
PASE*	179.0 ± 82.8	161.4 ± 80.1	164.1 ± 80.9	168.4 ± 84.0	169.1 ± 83.1	159.6 ± 78.1	159.6 ± 80.4	161.4 ± 81.0
Total energy intake (kcal/day)*	1274.7 ± 453.1	1323.2 ± 486.0	1473.4 ± 595.0	1511.9 ± 634.4	1298.3 ± 475.6	1321.4 ± 468.3	1449.3 ± 581.6	1501.1 ± 621.1

*OAI* Osteoarthritis Initiative, *ROA* radiographic knee osteoarthritis, *SxOA* symptomatic knee osteoarthritis, *E-DII* energy-adjusted dietary inflammatory index, *IQR* interquartile range

*All such values are mean ± standard deviation

As shown in Table 2, a higher E-DII score was associated with an increased risk of incident ROA in both the base model and fully adjusted model. Compared with subjects in Q1, those in Q4 had a higher risk of incident ROA with an odds ratio (OR) of 1.73 (95% confidence interval (CI) 1.15 to 2.62). Test for the linear trend of E-DII score with the risk of incident ROA was statistically significant (*P* for trend = 0.007). A similar association between E-DII score and incident SxOA also was observed. Subjects in Q4 had a significantly higher risk of incident SxOA than their counterparts in Q1 (OR = 1.43, 95% CI 1.16 to 1.76, *P* for trend = 0.001). When we fitted E-DII as a continuous variable in both the base model and fully adjusted model, the association of E-DII with ROA (OR from the fully adjusted model = 1.09, 95% CI 1.01, 1.17) and SxOA (OR from the fully adjusted model = 1.06, 95% CI 1.02, 1.11) remained statistically significant. When total knee replacement due to knee OA was counted as an incident case of either ROA or SxOA, the results did not change materially.

**Table 2:** Total effect of E-DII on incident ROA and incident SxOA

E-DII in quartile	Q1	Q2	Q3	Q4	*P* for trend*
**ROA**					
Median	− 3.9	− 2.7	− 1.3	0.9	
ROA knees^**#**^	48/896	53/892	66/892	65/892	
Model 1^†^	1.00 (ref)	1.12 (0.75, 1.68)	1.48 (1.00, 2.18)	1.43 (0.96, 2.11)	0.049
Model 2^‡^	1.00 (ref)	1.27 (0.84, 1.94)	1.53 (1.01, 2.32)	1.73 (1.15, 2.62)	0.007
**SxOA**					
Median	− 3.9	− 2.7	− 1.4	0.8	
SxOA knees^**#**^	218/1472	237/1470	260/1470	263/1468	
Model 1^†^	1.00 (ref)	1.12 (0.92, 1.37)	1.28 (1.05, 1.56)	1.37 (1.12, 1.68)	0.001
Model 2^‡^	1.00 (ref)	1.13 (0.92, 1.40)	1.27 (1.04, 1.56)	1.43 (1.16, 1.76)	0.001

*E-DII* energy-adjusted dietary inflammatory index, *BMI* body mass index, *OA* osteoarthritis, *PASE* Physical Activity in the Elderly Scale, *ROA* radiographic knee osteoarthritis, SxOA, symptomatic knee osteoarthritis

*Test for trend based on the variable containing the median value for each quartile

^**#**^Number of OA affected/total number of knees in each quartile of the dietary inflammatory index

^†^Model 1 adjusted for age (years), sex (men vs women), race (white vs non-white), and total energy intake (kcal/day)

^‡^Model 2 further adjusted for education (< college vs ≥ college), yearly income level (< 50,000 US$ vs ≥ 50,000 US$), tobacco use (non-smoker vs smoker), and physical activity (PASE, continuous)

The total, natural direct, and indirect effects of E-DII score on the risk of incident ROA mediated via BMI are shown in Table 3. BMI significantly mediated the effect of E-DII on incident ROA, comparing participants with an E-DII < median (i.e., 2.50) with those whose E-DII ≥ median (OR for indirect effect = 1.08, 95% CI 1.04 to 1.13), with a proportion of mediation of 20.4%. The direct effect of E-DII on incident ROA not through BMI was statistically significant (OR = 1.36, 95% CI 1.02 to 1.82). Similarly, the indirect effect (i.e., mediated effect) of E-DII on incident SxOA through BMI was 1.13 (95% CI 1.09 to 1.16) while the direct effect (OR = 1.16, 95% CI 1.01, 1.34) also was statistically significant. About 44.5% of the association between E-DII score and the risk of incident SxOA was mediated via BMI.

**Table 3:** Direct and indirect effects of E-DII on incident ROA and SxOA for mediation via BMI

E-DII	< 2.50	≥ 2.50
ROA	*n* = 894 persons	*n* = 892 persons
Total effect*	1.00 (ref)	1.47 (1.10, 1.97)
Direct effect	1.00 (ref)	1.36 (1.02, 1.82)
Indirect effect via BMI	1.00 (ref)	1.08 (1.04, 1.13)
Proportion of mediation via BMI	20.4%	
SxOA	*n* = 1471 persons	*n* = 1469 persons
Total effect*	1.00 (ref)	1.31 (1.13, 1.51)
Direct effect	1.00 (ref)	1.16 (1.01, 1.34)
Indirect effect via BMI	1.00 (ref)	1.13 (1.09, 1.16)
Proportion of mediation via BMI	44.5%	

*E-DII* energy-adjusted dietary inflammatory index, *BMI* body mass index, *ROA* radiographic knee osteoarthritis, *SxOA* symptomatic knee osteoarthritis

*Model adjusted for age (years), sex (men vs women), race (white vs non-white), total energy intake (kcal/day), education (< college vs ≥ college), yearly income level (< 50,000 US$ vs ≥ 50,000 US$), tobacco use (non-smoker vs smoker), and physical activity (PASE, continuous)

## Discussion

In this prospective US cohort, we found that a higher E-DII score was associated with an increased risk of incident ROA and SxOA after controlling for several potential confounders including total energy intake. The impact of the DII on incident knee ROA and SxOA was, in part, due to its effect on BMI.

Observational studies on single dietary components have failed to be corroborated in randomized trials. For example, supplements of vitamin E were ineffective while vitamin D did not achieve a clinically important effect on knee pain in OA compared with placebo [32]. However, people do not consume nutrients or foods in isolation. The importance of accounting for the effect of diet as a whole has been emphasized increasingly over the past several years [1, 2, 16, 33]. Using a dietary index to quantify the inflammatory potential of the whole diet may increase the robustness and validity in detecting the relation to disease [16]. The DII and, by logical extension, the E-DII account for a variety of nutrients and foods for which evidence indicates the ability to exert pro- or anti-inflammatory effect within a diet [23]. Our study, as an advance over previous observational studies focusing on single dietary components, is the first one to use a dietary index to examine the association between diet and incident knee OA. Our findings, if confirmed in other, larger prospective studies, may inform a prevention strategy, with more emphasis on the effect of diet as a whole, for this highly prevalent disabling disease.

There is evidence that a higher DII score increases the risk of weight gain and incident obesity, independent of total energy intake [7]. Thus, an intuitive question is: To what extent the DII-OA association is mediated via BMI with adjustment for total energy intake? Indeed, we found the effect of the E-DII on incident knee OA was substantially mediated through BMI as evidenced by a proportion of mediation of 20.4% for ROA and 44.5% for SxOA, which is independent of total energy intake. Weight loss reduces the risk of symptomatic knee OA [34]. Dietary strategies to lose weight have primarily focused on limiting total energy intake [35–37]. Our findings, along with those of previous studies [6, 7], suggest that a diet with low inflammatory potential may be synergistic in helping individuals to reduce the risk of incident knee OA. Because diet can affect inflammation through a variety of dietary parameters, its effect is not limited to restricting total energy intake. It is plausible to take inflammatory potential of diet into consideration in dietary strategies for people at high risk of developing knee OA. Nevertheless, observational studies can only provide empirical evidence; thus, randomized clinical trials may be warranted to test this hypothesis. However, it is important to understand the nuances of intervening in dietary behaviors [38].

Inflammation plays an integrated role in OA pathology [39, 40]. Diet regulates inflammation [3–5, 41, 42]. Inflammatory potential of diet, as quantified by DII and the E-DII, predicts circulating inflammatory markers such as IL-6, TNF-α, and CRP [22, 24–27]. It has been shown that these inflammatory markers are associated with knee pain and joint damage [43–45]. In our study, mediation analyses showed that the direct effect of the E-DII on incident knee ROA or SxOA was statistically significant, suggesting the association between E-DII score and the risk of knee OA may not be entirely accounted by its effect on BMI. The DII-OA relation also may be mediated via inflammatory markers. However, due to a lack of data on circulating inflammatory markers in OAI, we were not able to examine this potential causal pathway of DII-OA. Further studies are needed to address this question. These must be designed to elucidate the temporal order of effects, an important criterion for assessing causality [46, 47]. Furthermore, these studies must account for the knowledge that diet can exert a direct effect on inflammation (i.e., not mediated via BMI or adiposity) as well as its role in affecting energy balance [6, 48, 49].

There are notable strengths of this study. We used data from OAI, a well-executed multi-center longitudinal cohort study with a large sample. The exposure, mediator, and outcomes in our study were assessed using validated instruments and standard protocols. Central reading of knee radiographs blinded to the chronological order of follow-up with consensus adjudication is especially appreciated to improve the reliability of incident knee OA assessment. We were able to account for the intraclass correlation between knees within individuals using the GEE approach. This is important because individuals tend to get bilateral OA of the knee [50]. The DII/E-DII is a well-validated tool that can be applied in any population with a wide range of data source [23, 24]. The use of objective measurements of body weight and height reduces potential biases. Furthermore, E-DII scores were associated with both incident ROA and SxOA. Consistent findings lend additional validity to our study.

Our findings should be interpreted with caution. First, only 24 out of 45 food parameters were available for calculating E-DII scores in the OAI, resulting in a less-than-ideal assessment of the inflammatory potential of diet. However, the predictive ability of the DII has been shown to be relatively preserved in calculations using < 30 parameters [24]. Moreover, the FFQ covered a majority of dietary components that have been related to OA in the literature [14, 15], with eight out of nine pro-inflammatory food parameters accounted for in calculating DII/E-DII [23]. Second, given dietary intakes were queried only once at the baseline visit, misclassifications may occur due to natural, seasonal fluctuations in dietary intake and secular trends in dietary change. Nevertheless, such exposure misclassification, if occurred, is likely to be non-differential and would bias the effect estimate towards the null. Third, physical activity was assessed only using the self-reported PASE questionnaire at baseline visit in the OAI, which may not be as accurate and reliable as that by objective measures. However, the PASE questionnaire has been validated for the assessment of physical activity among adults with knee pain and disability [29]. Finally, our findings may not be fully generalizable to other populations due to the selection of participants who were at high risk of knee OA in OAI. Other factors, such as genetic background and environmental exposures, also should be taken into consideration when interpreting our findings.

## Conclusions

Greater dietary inflammatory potential, as expressed as the E-DII, was associated with an increased risk of developing knee OA in this prospective US cohort. Such an association was mediated by BMI independent of total energy intake, suggesting an integrated role of inflammatory potential in dietary strategy for people at high risk of knee OA.

## Data Availability

The datasets generated and/or analyzed during the current study are available in the OAI repository, https://nda.nih.gov/oai.

## Abbreviations

OA: Osteoarthritis
BMI: Body mass index
DII: Dietary inflammatory index
SxOA: Symptomatic knee OA
OAI: Osteoarthritis Initiative
FFQ: Food Frequency Questionnaire
CRP: C-reactive protein
IL: Interleukin
TNF: Tumor necrosis factor
E-DII: Energy-adjusted DII
KL: Kellgren and Lawrence
ROA: Radiographic knee OA
PASE: Physical Activity Scale for the Elderly
GEE: Generalized estimating equations
OR: Odds ratio
CI: Confidence interval
